# Neurological symptoms and signs in thyroid disease

**DOI:** 10.1186/1756-6614-8-S1-A25

**Published:** 2015-06-22

**Authors:** Mariusz Stasiolek

**Affiliations:** 1 Department of Neurology, Polish Mother’s Memorial Hospital – Research Institute, Lodz, Poland

## 

Numerous complex regulatory mechanisms influence the development and function of the peripheral and central nervous system. Among them, hormones belong to the most potent regulatory factors. Various particles known for their hormonal activity serve as neurotransmitters. Additionally, hormones secreted systemically modulate the function of the nervous system both on the brain level and in peripheral organs. Thyroid function has been shown to play a crucial role in the proper cognitive development but also in many other aspects of nervous system activity, in mechanisms involving direct interaction with intrinsic regulatory circuits or indirectly by systemic effects exerted e.g. on the circulatory system or metabolic pathways. Due to these close relations with the nervous system function, disturbances of thyrometabolic state are associated with a vast spectrum of neurological signs and symptoms including: mood and cognitive disorders, headache, ophthalmoplegia, tremor and other movement disorders, muscle weakness etc. Both hyper- and hypothyroidism may cause psychiatric symptoms like depressive or anxiety disorder, memory deficits, executive inability and even psychosis. The severe decompensated hypothyroidism may result in myxoedema coma - a life-threatening condition with sequentially progressing encephalopathic symptoms. Steroid-responsive encephalopathy associated with autoimmune thyroiditis (SREAAT) represents another form of encephalopathic disorder associated with thyroid disease and causing potentially serious clinical complications. In the periphery, the thyrometabolic disturbances may affect muscle function resulting in subjective tiredness and low exercise tolerance and in some cases (especially in hypothyroidism) in objective myopathic signs. Also peripheral nervous system may be affected, mainly in hypothyroid patients, with greater tendency to develop peripheral polyneuropathy and entrapment neuropathies such as carpal tunnel or Guyone canal syndromes.

Importantly, the autoimmune thyroid disease has been shown to coexist with other autoimmune processes which may potentially cause neurological symptoms such as myasthenia, Guillain-Barre syndrome or pernicious anaemia. Such conditions have to always be taken in consideration as differential diagnoses in patients presenting with neurological signs and symptoms associated with thyroid disease.

